# Reviewer acknowledgement 2012

**DOI:** 10.1186/1476-0711-12-5

**Published:** 2013-04-05

**Authors:** Hakan Leblebicioglu

**Affiliations:** 1 Department of Infectious Diseases and Clinical Microbiology, Faculty of Medicine, Ondokuz Mayis University, Samsun, Turkey

## Abstract

**Contributing reviewers:**

*Annals of Clinical Microbiology and Antimicrobials* would like to thank the following colleagues for their assistance with peer review of manuscripts for the journal in 2012.

## 

Iqbal Ahmad

India

Edith Ajaiyeoba

Nigeria

Patrick Eberechi Akpaka

Trinidad And Tobago

Farrukh Aqil

United States of America

Ferhat Arslan

Turkey

Rehab Ashour

Egypt

Daniela Badescu

Romania

Thiagarajan Balasubramanian

India

Fernando Baquero

Spain

Yasar Bayindir

Turkey

Ciomar A Bersani-Amado

Brazil

Giuseppe Blaiotta

Italy

Sonia Borrell

Switzerland

Lyudmila Boyanova

Bulgaria

Emilia Canton

Spain

Stephane Chevaliez

France

Misca Corina

Romania

Marialaura Corrente

Italy

Omer Coskun

Turkey

Everardo Curiel-Quesada

Mexico

Bruno Da Rocha-Azevedo

United States of America

Olivier Dauwalder

France

Frank Deleo

United States of America

Corine Delsing

Netherlands

Veronique Dubois

France

Kalsoom Farzana

Pakistan

Mohammad Feizabadi

Iran

Masaki Fujita

Japan

Moreno Galleni

Belgium

Victor Gannon

Canada

Claudiu Georgescu

United States of America

Giovanni Gherardi

Italy

Anupam Ghosh

India

Ilhami Gulcin

Turkey

Sibel Gundes

Turkey

Azar Hadadi

Iran

Thomas Herchline

United States of America

Duane Hospenthal

United States of America

Wensi Hu

Taiwan

M. Idrees

Pakistan

Nejat Isik

Turkey

Susie Jerwood

United Kingdom

Uner Kayabas

United Kingdom

Noha Khalaf

Egypt

Muhammad Khurram

Pakistan

Jan Kielstein

Germany

Kwang-Sik Kim

United States of America

Marlise Klein

United States of America

Milan Kojic

Serbia

Bochra Kouidhi

Tunisia

Rajesh Kumar

India

Andrea Laukova

Slovakia

Chiou-Feng Lin

Taiwan

Nilton Lincopan

Brazil

Ming-Li Liou

Taiwan

Po-Yu Liu

Taiwan

Elisabete Machado

Portugal

Eric Macy

United States of America

Runner R. T. Majinda

Botswana

Helen Maltezou

Greece

Gian Luigi Mariottini

Italy

Fatih Matyar

Turkey

Maryam Memarian

Iran

Sina Mobasherizadeh

Iran

Eiman Mokaddas

Kuwait

Alexandra Moura

Portugal

Maysaa Nile

Egypt

Yoshikazu Nishikawa

Japan

Lutfiye Oksuz

Turkey

Ellis Owusu-Dabo

United Kingdom

Resat Ozaras

Turkey

Manmohan Parida

India

Nsirimobu Paul

Nigeria

Ferenc Peles

Hungary

Tom Petro

United States of America

Gabriella Piatti

Italy

Iveta Placha

Slovakia

Roberto Posada

United States of America

Cosimo Prantera

Italy

K. V. Ramana

India

Karam Ramotar

Canada

Rabia Ramzan

Germany

Guy Richards

South Africa

Louise Rollins-Smith

United States of America

Victor D Rosenthal

Argentina

Maria Pilar Ruiz

Spain

Helio Sader

United States of America

Horieh Saderi

Iran

Sreenivasan Sasidharan

Malaysia

Don Schaffner

United States of America

Tim Schuurman

Netherlands

Gowhar Shafi

Sweden

Surender K Sharma

India

Li Shen

China

Bidya Shrestha

Nepal

Rubina Siddiqui

Pakistan

Oguz Resat Sipahi

Turkey

Aijaz Soomro

Pakistan

Damien Stark

Australia

Ivana Strahinic

Serbia

Tsuyoshi Sugiyama

Japan

Hare Krishna Tiwari

India

Selma Tobudic

Austria

Athanassios Tsakris

Greece

Aysel Ugur

Turkey

Ebru Us

Turkey

Haluk Vahaboglu

Turkey

Babette van Hees

Netherlands

Luca A. Vitali

Italy

Cyrus Githaiga Wagate

Kenya

Imen Wahabi

France

Congyi Wang

United States of America

Christina Welinder-Olsson

Sweden

Hao Wen

China

Gottfried Wilharm

Germany

Qingmei Xie

China

Jin Xu

China

Aysegul Yagci

Turkey

Hava Yilmaz

Turkey

Mesut Yilmaz

Turkey

Sang Sun Yoon

Korea South

Hu Zhu

China

Guo Ying Zuo

China

